# Isolation and characterization of strictly anaerobic cellulolytic rumen bacterial species from Sahiwal cattle

**DOI:** 10.5455/javar.2024.k740

**Published:** 2024-03-07

**Authors:** Muhammad Ashiqul Alam, Md. Jannat Hossain, M. Sohidullah, Md. Shahidur Rahman Khan, Khan Md. Shaiful Islam

**Affiliations:** 1 Department of Microbiology and Hygiene, Bangladesh Agricultural University, Mymensingh, Bangladesh; 2 Department of Microbiology and Public Health, Khulna Agricultural University, Khulna, Bangladesh; 3 Department of Animal Nutrition, Bangladesh Agricultural University, Mymensingh, Bangladesh

**Keywords:** Bacteria, cattle, cellulolytic, rumen, *Ruminococcus* spp

## Abstract

**Objective:**

To isolate and characterize cellulolytic rumen bacteria from the rumen of Sahiwal cattle using rumen bacterial inoculum.

**Materials and Methods:**

The ruminal liquid was kept at an optimal pH of 6.9 and a redox potential of less than −300 mV while being incubated anaerobically at 39°C in a medium containing rumen fluid glucose cellobiose agar. By using the Hungate technique, the organisms were detected based on their morphological, physiological, biochemical, and molecular testing.

**Results:**

The findings revealed that the isolated *Ruminococcus albus*, and *Ruminococcus flavifaciens* were obligate anaerobic, generally Gram-positive, nonmotile cocci or rod, single or pair, occasionally short chain, producing yellow pigment when grown on cellulose, and having a clear zone around the colonies. Both isolate fermented sugars such as cellobiose, glucose, and lactose, as well as decomposed xylan. The results also showed that the isolates recognized as *Ruminococcus* spp., a cellulolytic rumen bacterium, were catalase-negative, indole-negative, and gelatin liquefaction-positive.

**Conclusion:**

Isolation and characterization of *Ruminococcus* spp. may be helpful for Bangladesh in reducing the cost of producing poultry feed and circumventing restrictions on rice bran use. We can also develop more efficient and long-lasting plans to enhance poultry performance and feed efficiency, as well as increase the nutritional value of rice bran used as broiler feed, by understanding how various *Ruminococcus* spp. function in this process.

## Introduction

A variety of microorganisms, such as bacteria, protozoa, fungi, and viruses, live in the rumen, which is a complex and dynamic habitat [1,2]. These microbes help in the digestion of complex plant polysaccharides and proteins in cattle and other ruminants’ rumen, converting them into simpler compounds that animals can ingest and use for energy and nutrients [3]. Rumen bacteria can control some of the most important metabolic activities, such as the breakdown of plant cell walls, the production of volatile fatty acids (VFAs), and the control of the ruminal microbial population [4]. *Fibrobacter, Ruminococcus, Prevotella,* and* Streptococcus* are the major genera of rumen bacteria [5]. The species belonging to these genera have distinct and complementary activities in the rumen, adding to the overall effectiveness and efficiency of the digestive process.

Numerous bacteria are found in the rumen of cattle that enable the biotransformation of nutrients into an energy source for cattle [6]. The bacteria belonging to the genus *Ruminococcus* are Gram-positive cellulolytic and are frequently found in the rumen of cattle and other ruminants. Some of the most prevalent cellulolytic rumen bacteria are *Ruminococcus flavefaciens*, *Ruminococcus albus*, *Bacteroides succinogenes,* and *Buryrivibrio fibrisolvens* [7]. These Gram-positive cellulolytic bacteria are mandatory for the breakdown of plant polysaccharides and the production of VFAs, which help ruminant animals get energy [3].

By producing a variety of carbohydrate-active enzymes such as endo-1,4-glucanases, xylanases, and cellulases, *Ruminococcus* spp. can break down plant cell walls, which can decrease fiber and increase crude protein [8]. To increase feed efficiency and performance in poultry, an alternate strategy is the use of rumen bacterial inoculum as a feed additive. Though *Ruminococcus* spp. is engaged in the breakdown of plant cell walls, it also produces lactic acid, ethanol, and various short-chain fatty acids, among other metabolic byproducts [9]. These substances are helpful to keep the rumen’s pH and redox balance in check and restrain the growth of harmful pathogens. The prevalence and quantity of different *Ruminococcus* spp. in the rumen can be affected by the type and quality of the feed being ingested, the presence of other rumen microbes, the overall health and productivity of the animal, and other factors [3,10]. By preserving a balanced and diversified rumen microbial population, *Ruminococcus* spp. and other rumen bacteria help in the efficient and effective assimilation of feed in cattle, as well as the overall health and productivity of the animal [3].

This study was undertaken to find and detect the presence of Gram-positive, strictly anaerobic *Ruminococcus* spp. in bovine rumens, which may be helpful for Bangladesh in reducing the cost of producing poultry feed, circumventing restrictions on rice bran use, and understanding how various *Ruminococcus* spp. function in this process. This can aid in the development of more efficient and long-lasting plans for enhancing poultry performance and feed efficiency.

## Materials and Methods

### Ethical approval

Approval of the study protocol was obtained from the Ethical Committee of Bangladesh Agricultural University (AWEEC/BAU/2020-57).

### Study period and location

This research aimed to isolate, recognize, and characterize specific bacteria found in rumen contents by molecular analysis. In the year 2020, this research work was carried out in the Department of Microbiology and Hygiene at Bangladesh Agricultural University (BAU), Mymensingh.

### Collection of rumen liquid samples

The samples of rumen fluids were taken from 2-year-old healthy Sahiwal cattle (*Bos taurus*) using a polyvinyl pipe through a fistula and into pre-gassed flasks. The flask was kept within a thermally insulated bucket filled with warm water heated to 40°C during transportation to maintain an airtight seal and a temperature of 39°C. The samples were then gassed for 10 min with carbon dioxide (CO_2_) before being immediately moved to a controlled anaerobic workstation that was saturated with oxygen-free nitrogen, CO_2_, and hydrogen gas in an 80:10:10 ratio and kept at a temperature of 39°C and a humidity level of 70%. By swirling the samples for 10 min with a stir stick, the samples were homogenized to liberate bacteria linked with feed particles and then processed for further analysis. We collected samples from Shahjalal Animal Nutrition Lab, BAU.

### Identification of rumen bacteria

Using anaerobic hungate techniques of serial dilutions by anaerobic diluent solution, where the anaerobic condition was confirmed by crystal clear color from violet after autoclaving and repeated tubing, in particular rumen fluid glucose cellobiose agar (RGCA) broth media, rumen bacteria were isolated from the contents of cattle rumens [11,12]. In addition, RGCA was used to evaluate the morphological and cultural characteristics of rumen bacteria under anaerobic circumstances using staining techniques.

### Gram’s staining technique

Longer safranin staining (3–5 min) was done in addition to the normal Gram stain method. The research was conducted using the methodology outlined by Merchant and Packer [13].

### Congo red staining

Cellulolytic rumen bacteria were identified using the Congo red staining technique. This technique detects β-D-glucan degradation and offers a quick and accurate screening test for *Ruminococcus* species cellulolytic bacteria [14].

### Fermentation of sugars with starch

Mineral solution No. 1 (15%), mineral solution No. 2 (15%), resazurin (0.0001%), trypticase (1.5%), yeast extract (0.5%), rumen fluid (10%), sodium carbonate (0.4%), and cysteine hydrochloride (0.05%) were the ingredients that were included in the basal medium to identify rumen bacteria. The medium was utilized to quantify the liquefaction of gelatin as well as the formation of acid from glucose, d-xylose, and cellobiose. Starch fermentation was discovered using a different medium that contained the basal medium plus 0.1% soluble starch. The ruman fluid glucose cellobiose broth medium was identical to the cellulose digestion medium, but it contained cellobiose (0.01%) and cellulose (0.2%) instead of agar and glucose. In anaerobic conditions, CO_2_ gas was used to prepare all media [15].

### Fermentation reactions with sugars

For physiological tests, isolated bacteria were inoculated with 48–72 h of cultures in basal media. The cultures were derived from the broth culture of the RGCA medium. To determine how much acid was produced from d-xylose and cellobiose, bromocresol green was added. According to Bryant and Burkey [16], a glass electrode pH meter was applied to measure the generation of acid from glucose.

### Starch hydrolysis

The detection of starch hydrolysis was done by adding Gram’s iodine solution to the cultures after a week when the color change occurred. Bryant and Burkey described how to use this technique [16].

### Indole production test

Each test tube included 5 ml of tryptophan broth, which was autoclaved at 121°C and 15 PSI (pounds per square inch). The tubes were sterile and injected with a small amount of the experimental bacteria from the 24-h pure culture. The tubes were kept in anaerobic conditions for 48 h at 37°C. To produce indole, five drops of Kovac’s reagent were injected straight into the tubes [17].

### Catalase test

On a petri plate, a microscopic slide was put. Then, onto the microscopic slide, a few bacteria from the 24-h pure culture were inoculated using a sterile inoculating loop. Using a dropper, a drop of 3% H_2_O_2_ was applied to the microscopic slide where the organism was present and observed for any instantaneous bubble development [18].

### Gelatin liquefaction test

To test the gelatin’s ability to liquefy, 5% gelatin was added to the basal medium. It was made using an anaerobic process using CO_2_ gas. A rubber stopper holding nutrient gelatin medium was used to inject a 48-h-old inoculum of test bacteria into the serum container using an insulin syringe. After cultures were incubated for a week, Bryant and Burkey tested their capacity to liquefy gelatin [16].

### Molecular detection of isolated rumen bacteria

The Wizard^®^ Genomic DNA Purification Kit (Promega, USA) was used to purify the rumen bacterial DNA [19]. These isolates’ genomic DNAs were purified and extracted using a genomic DNA extraction kit (Promega^®^). Using a species-specific primer, the polymerase chain reaction (PCR) procedure was carried out as previously described [20]. Using the primers listed in Table 1, PCR was carried out for the identification of the bacterial species using the 16S rRNA gene. Positive control was not used because it was the first study of this type from Bangladesh describing the isolation, PCR detection, and characterization of *Ruminococcus* spp., but negative control was used. The amplified PCR products were separated by electrophoresis in 2% agarose gel at 100 V for 30 min. After that, staining was done with ethidium bromide in TAE buffer and then seen under a UV transilluminator.

## Results

From the rumen contents of the Sahiwal cattle (*B. taurus*) in Bangladesh, ruminal bacteria, including *R. albus* and *R. flavefaciens* were identified and described.

### Cultural and morphological characteristics of the isolated bacteria

**Figure 1.**
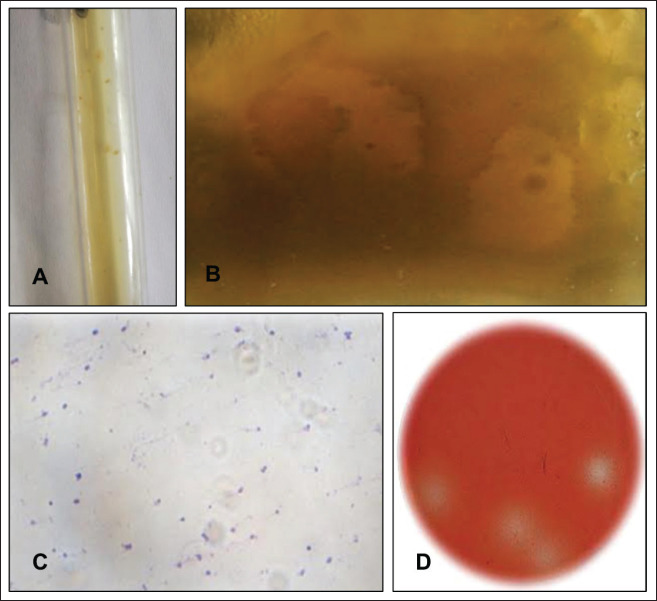
(A) Isolated *Ruminococcus* spp. showed a yellowish picnotic colony, (B) *Ruminococcus* spp. showed a clear zone around the colony, (C) *Ruminococcus* spp. at 100× magnification, and (D) *Ruminococcus* spp. showed a clear zone around the colony by Congo red stain.

RGCA was used in anaerobic conditions to examine the morphological and cultural characteristics of *R. albus* and *R. flavefaciens*. Small, picnotic, yellowish colonies of these only anaerobic bacteria were visible on RGCA (Fig. 1). Ruminal bacteria were identified by the gram’s staining method as coccoid, Gram-positive, single, and paired (Fig. 1). A distinct zone was also visible around the colony in the Congo-red staining (Fig. 1).

The screening process for cellulolytic bacteria like *Ruminococcus* spp. used specific media, such as rumen-specific carboxymethyl cellulose (CMC) Congo-red agar, to obtain 8 out of 12 bacterial isolates as an indicator for β-D-glucan degradation. The morphology, staining, and cultural properties of the rumen bacteria were examined in appropriate culture media. All of the 12 isolates shared a striking resemblance in terms of morphology.

**Table 1.** Species-specific 16S rRNA gene primer sequences used in this research.

Bacterium	Primer name	Sequence (5’–3’)	Product size (bp)	References
*R. albus*	Ra1281f	CCCTAAAAGCAGTCTTAGTTCG	175	[20–22]
	Ra1439r	CCTCCTTGCGGTTAGAACA		
*R. flavefaciens*	Rf154f	TCTGGAAACGGATGGTA	295	[20,21]
	Rf425r	CCTTTAAGACAGGAGTTTACAA		

**Table 2.** Results of sugar fermentation, starch hydrolysis, indole test, and catalase and gelatin liquefaction test on different bacterial isolates.

Bacterial isolates	Indole	Catalase	Starch hydrolysis	Gelatin liquefaction	Sugar fermentation		
					Glucose	Xylose	Cellobiose
RA1	-	-	+	+	+ (g)	+ (g)	+ (g)
RA2	-	-	+	+	+ (g)	+ (g)	+ (g)
RA3	-	-	+	+	+ (g)	+ (g)	+ (g)
RA4	-	-	+	+	+ (g)	+ (g)	+ (g)
RA5	+	-	+	+	+ (g)	+ (g)	+ (g)
RA6	-	+	+	+	+ (g)	+ (g)	+ (g)
RF1	-	-	+	+	+ (g)	+ (g)	+ (g)
RF2	-	-	+	+	+ (g)	+ (g)	+ (g)
RF3	-	-	+	+	+ (g)	+ (g)	+ (g)
RF4	-	-	+	+	+ (g)	+ (g)	+ (g)
RF5	+	+	+	+	+ (g)	+ (g)	+ (g)
RF6	+	-	+	+	+ (g)	+ (g)	+ (g)

“+” indicates the presence of acid and “g” indicates the presence of gas.

### Biochemical characterization of isolated bacteria

**Figure 2.**
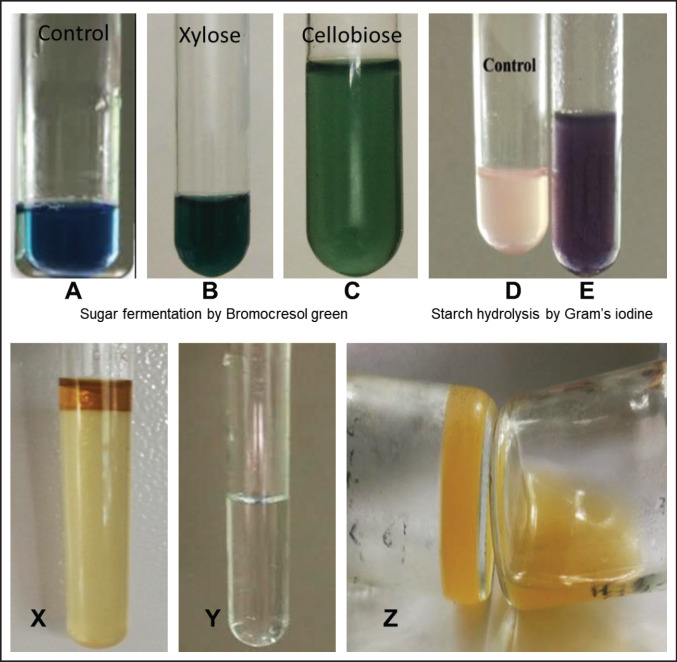
Suger fermentation by bromocresol green and indole test. (A–C) Sugar was fermented by using bromocresol green in xylose and cellobiose. Acid production was indicated by changing the color from blue to green. (A) Control, (B) xylose, (C) cellobiose, (D–E) starch hydrolysis by Gram’s iodine, (D) control, and (E) starch hydrolysis using Gram’s iodine changes the color from pink to violet. (X–Y) Indole test, (X) Indole test (presence of yellowish band), (Y) Catalase test (No bubble produced), and (Z) gelatin liquefaction.

To determine the exact type of bacteria in this study, biochemical tests such as sugar fermentation, Indole, catalase, gelatin liquefaction, and so on, were carried out (Table 2). The bacteria fermented the sugars glucose, cellobiose, and d-xylose into gas and acid, as seen by the color shift from blue to green (Fig. 2). The tests for indole and catalase were negative (Fig. 2). However, the tests for starch hydrolysis and gelatin liquefaction were successful, as in the case of starch hydrolysis, the color was shifted from pink to violet (Fig. 2).

After 72 h of fermentation, all 12 isolates were demonstrated to drop pH from 6.97 to 6.60 or lower, as seen by the culture medium transition from blue to green. The media’s color change and the increasing pressure inside the cultured vials allowed us to recognize the generation of acid or gas in this investigation, but the acid or gas in the culture systems was not identified or measured.

### Molecular detection of isolated bacteria

**Figure 3.**
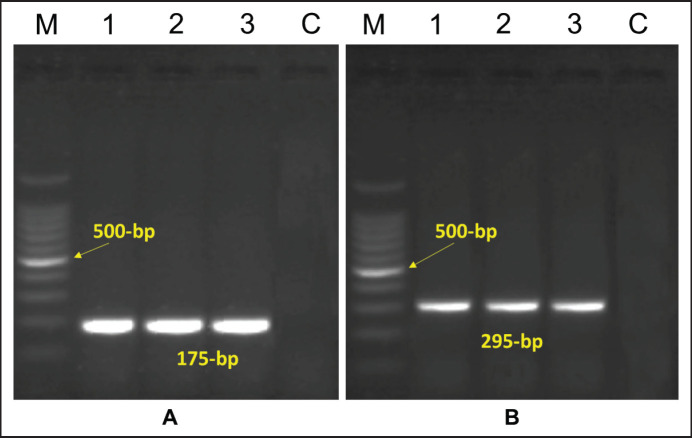
PCR identification of *R. albus* and *R. flavefaciens.* (A) Identification of *R. albus* from rumen contents. 175 bp were seen as a positive size. *Ruminococcus albus* suspected in lanes 1 through 3. (B) Identification of *R. flavefaciens* from rumen contents. (C). Control without DNA, *M*- 100 bp ladder. 295 bp were seen as a positive size. *Ruminococcus flavefaciens* suspected in lanes 1 through 3.

The bacterial species was determined using the molecular techniques of *Ruminococcus* spp. As per Table 3 and Figure 3, 12 isolates were thought to be *Ruminococcus* spp. For species identification, every isolate was selected.

Four of the 12 isolates (RA1, RA2, RA3, and RA4) were shown to be positive for *R. albus* through the identification of distinct bands at 175 base pairs (bp) using the Ra1281f and Ra1439r primers (Fig. 3). Using the Rf154f and Rf425r primers, specific bands at 295 bp were discovered to be positive for *R. flavefaciens* in the other four samples, such as RF1, RF2, RF3, and RF4 (Fig. 3). Unknown isolates were designated as RA5, RA6, RF5, and RF6.

## Discussion

More than 200 bacterial species per milliliter are present in rumen fluid [21]. Rumen bacteria mostly consist of *Fibrobacter succinogenes*, *R. flavefaciens*, and *R. albus*. We only detected and described *R. albus* and *R. flavefaciens* from the ruminal contents of the Sahiwal cattle (*B. taurus*) in Bangladesh.

Small picnotic yellowish colonies of *Ruminococcus* spp. were visible on RGCA (Fig. 1). Gram’s staining method described *Ruminococcus* spp. as coccoid, Gram-positive, single, and paired (Fig. 1). Other researchers also reported *Ruminococcus gnavus* as coccoid and Gram-positive [22]. In the Congo-red staining, we observed a distinct zone around the colony (Fig. 1).

**Table 3.** Results of isolated rumen bacteria by PCR.

Bacterial isolates	Band size	Remarks
RA1	175	*R. albus*
RA2	175	*R. albus*
RA3	175	*R. albus*
RA4	175	*R. albus*
RA5	-	Unidentified
RA6	-	Unidentified
RF1	295	*R. flavefaciens*
RF2	295	*R. flavefaciens*
RF3	295	*R. flavefaciens*
RF4	295	*R. flavefaciens*
RF5	-	Unidentified
RF6	-	Unidentified

Rumen-specific CMC Congo-red agar was used for *Ruminococcus* spp. to obtain 8 out of 12 bacterial isolates as an indicator for β-D-glucan degradation. An almost similar result was reported in another study [23]. All 12 isolates shared a striking resemblance in terms of morphology, according to earlier descriptions of *Ruminococcus* spp. [7]. Though *Ruminococcus* spp. were isolated in this study, rumen contents also contain *Pseudomonas aeruginosa*, *Bacillus*, *Micrococcus*, and *Streptococcus* species, which are involved in the breakdown of cellulose and resemble the cellulolytic *Ruminococcus* spp. bacteria previously reported by another study [14].

*Ruminococcus* spp. shifted the color from pink to violet through the fermentation of sugars such as glucose, cellobiose, and d-xylose into gas and acid (Fig. 2). A more or less similar result was also reported by another study [24]. Many researchers have recorded that, in comparison with other cellulolytic strains, *R. albus* could ferment many types of carbohydrates, mainly cellulose, glucose, xylose, and mannitol [25]. Though tests such as indole and catalase were negative (Fig. 2), starch hydrolysis and gelatin liquefaction tests were positive (Fig. 2). *Ruminococcus* spp. has previously been reported as having comparable biochemical characteristics in Bergey’s manual of systemic bacteriology and Bryant and Burkey [16].

All the bacterial isolates showed a drop in pH from 6.97 to 6.60 or lower through the culture medium’s transition from blue to green after 72 h of fermentation. The three main ruminal cellulolytic bacteria, such as *F. succinogenes*, *R. albus*, and *R. flavefaciens*, were implicated in the production of volatile nutrients in earlier research [26]. *Ruminococcus flavefaciens* produced succinate, acetate, ethanol, and format in significant quantities during the fermentation of cellulose and cellobiose. However, it produces less CO_2_ or hydrogen [27].

In this study, 12 isolates were thought to be *Ruminococcus* spp. (Table 3; Fig. 3). Ra1281f and Ra1439r primers detected four isolates, namely RA1, RA2, RA3, and RA4, as positive for *R. albus* by showing distinct bands at 175 bp. The other four samples, RF1, RF2, RF3, and RF4, were positive for *R. flavefaciens* by showing specific bands at 295 bp using the Rf154f and Rf425r primers. *Ruminococcus albus*, *R. flavefaciens*, and *F. succinogenes* are three species of ruminal cellulolytic bacteria that have been previously identified in ruminal fluid, where *R. albus* is typically the most prevalent [28].

Isolated cellulolytic *Ruminococcus* spp. from the rumen liquid of cattle could improve crude protein in urea-treated fermented rice bran (UFRB) (18.43%) than control rice bran (RB) (14.42%), but decrease crude fiber in all the treated groups than RB (12.57%), where the least crude fiber was recorded as 9.92% in UFRB (*p* < 0.05). In addition, the percentage of phytate-P was decreased in UFRB (1%) compared to RB (1.12%) through anaerobic fermentation of rice bran [29,30].

## Conclusion

This study reveals *R. flavefaciens* and *R. albus* as the two main species present in the rumen of cattle in Bangladesh, according to the collected data using Hungate techniques of culture enumeration and PCR. These species are the first to be identified in Bangladesh. *Ruminococcus albus* is increasingly being employed for the fermentation of rumen bacteria to determine its association with changes in RB’s nutritional value because it is the most prevalent species among rumen cellulolytic bacteria. Isolation and characterization of *Ruminococcus* spp. may be helpful for Bangladesh in reducing the cost of producing poultry feed and circumventing restrictions on rice bran use.

## List of abbreviations

BAU, Bangladesh agricultural university; CMC, carboxy methyl cellulose; PCR, polymerase chain reaction; PSI, pounds per square inch; RB, control rice bran; RGCA, rumen fluid glucose cellobiose agar; TAE, Tris-acetate-EDTA; UFRB, urea treated fermented rice bran; VFAs, volatile fatty acids.
